# Psychological sense of community as mediator and growth mindset as moderator in the impact of institutional integrity and perceived teacher support on student thriving: Evidence from private universities in China

**DOI:** 10.1371/journal.pone.0312338

**Published:** 2024-12-31

**Authors:** Yujun Jiang, Huying Liu, Zhiqun Ouyang, Meng Xie, Shihang Wei

**Affiliations:** 1 School of Language and Culture, Swan College, Central South University of Forestry and Technology, Changsha, China; 2 School of Foreign Languages, Hunan International Economics University, Changsha, China; 3 School of Education, Baoshan University, Baoshan, China; 4 Graduate School, Stamford International University, Bangkok, Thailand; Alexandru Ioan Cuza University of Iasi, Faculty of Philosophy and Social-Political Sciences, ROMANIA

## Abstract

Previous research may have focused more on the direct rather than indirect effects of psychological characteristics on student success. This study explored the mediating effect of psychological sense of community and the moderating effect of growth mindset on the impacts of institutional integrity and perceived teacher support on student thriving in academic, interpersonal, and psychological domains. The hypothesized research model was proposed based on the Thriving Model, and the study was carried out in the setting of private universities in China. This study employed a partial least squares structural equation modeling (PLS-SEM) approach, obtaining a total of 1792 valid questionnaire responses through an online survey. The research highlights that psychological sense of community plays a crucial role in mediating the impact of institutional integrity and perceived teacher support on student thriving. In addition, it was found that growth mindset positively moderates the impact of institutional integrity and perceived teacher support on student thriving. Some implications and recommendations for teachers, administrators, and researchers in relevant fields were provided.

## 1. Introduction

The psychological or spiritual characteristics of university students have received much attention from academics since the mission of higher education is holistic education [1]. A university is believed to provide adequate external support for students, enrich their internal experiences on campus, and help improve their comprehensive achievements, preparing them for future civic engagement [1]. Student thriving is the ideal framework to comprehensively examine the growth of college students, as it not only addresses their academic and social development but also highlights their psychological development [2]. Several external contextual aspects on campus, such as institutional integrity, student-faculty engagement, and the student’s internal psychological elements, including their psychological sense of community and growth mindset, contribute to student thriving [3]. These contributors are malleable, dynamic, and intervenable under different educational contexts [4], making the model ideal for studying student success at private higher institutions in China.

Private institutions in China have experienced significant growth in recent decades and have become an essential component of the country’s higher education system [5]. While these institutions mostly rely on self-financing, they are required to fulfill the educational requirements of students at lower academic levels seeking higher education, as well as the demands of Chinese society for a diverse workforce [6]. Presently, their primary focus is on the enrollment and job rates of graduates rather than the campus experience or holistic development of students; some of them are even regarded as “diploma mills” [7]. Private universities would exaggerate their admissions process, thus making it difficult to keep promises to the missions and goals reflected through their administration policy and educational practice [8]. Private universities often undervalue their teachers, which can undermine students’ trust in the teacher-student relationship. Students tend not to think that teachers are necessarily the best available resource when they need help [9]. Furthermore, students at Chinese private universities often exhibit a lower level of growth mindset or sense of belonging, partly due to their poor academic performance or their lack of ability to overcome challenges. Additionally, falling behind in their studies and failing to attend public universities can potentially undermine their spiritual beliefs in higher education.

In order to enhance the overall thriving of students at private universities in China, it is advisable to explore students’ psychological or spiritual well-being on campus, as well as their perception of support from the university and faculty members, and to scrutinize their respective roles. There has been much academic research on the direct contributors to student thriving, including students’ psychological aspects and university climate factors. However, more research is needed on the indirect effects of psychological or spiritual factors on the relationship between university support and student thriving. The objective of this study was to examine and validate the role of psychological sense of community as a mediator in the connection between institutional integrity, perceived teacher support, and student thriving. Furthermore, the study sought to investigate how the concept of growth mindset moderates the effect of institutional integrity and perceived teacher support on student thriving.

Schreiner proposed student thriving, drawing from Keyes’ flourishing theory and incorporating Eaton and Bean’s psychological retention model and Braxon and Hirschy’s persistence model [10]. The holistic approach assesses students’ experiences and accomplishments on campus in a comprehensive manner, encompassing their intellectual, social, emotional, and psychological aspects. It primarily focuses on their academic performance, interpersonal relationships, and psychological well-being [11]. The research findings have identified several factors that contribute to student thriving, including university support and students’ on-campus experiences. These factors include institutional integrity, sense of community, student-faculty interaction, growth mindset, campus involvement, and spirituality. It has been observed that these factors may vary in different contexts [12]. Various student demographics and populations, including diverse racial groups and transfer students [13], community college students [14], students from faith-based campuses [15], and graduate students [16], may have varied pathways to thriving. Therefore, it is necessary to determine the pathways to the thriving of students from Chinese private universities by doing empirical studies.

Community psychologist Sarason proposed psychological sense of community in the 1970s [17]. Later, McMillan and Chavis described it as “a feeling that members have of belonging, a feeling that members matter to one another and to the group, and a shared faith that members’ needs will be met through their commitment to being together” [18]. Extensive research in higher education consistently confirms that psychological sense of community has a clear correlation with both student social connectivity and academic success [2], their personal development [11], student satisfaction [19], perceptions of the institution [20], and psychological health [21]. For example, Schreiner et al. Adid a study involving 2,889 college students [12]. They discovered that psychological sense of community accounted for 32% of the variance in student thriving. The impact of psychological sense of community may vary depending on the student cohorts [22], and students from private universities in China tend to be more psychologically dependent [23]. The purpose of this study is to understand how students’ psychological sense of community affects their thriving in the context of private universities in China. The following hypothesis was formulated based on the above literature review:

**H1:** Psychological sense of community among students in Chinese private universities positively affects student thriving.

Institutional integrity is defined as the alignment between the institution’s stated mission and its actual behavior. This means that institutions fulfill their promises by adhering to their missions and goals through their administrative policies and educational practices [24]. It reflects students’ perceptions of “the degree of congruence between the espoused mission and goals of a college or university and the actions of administrators, faculty, and staff” [25]. The extent to which institutional practices reflect its mission is an indicator of students’ satisfaction [26], personal development and academic achievements on campus [4], and adjustment and engagement in the university community [24]. For example, first-generation students greatly benefit from their achievements by perceiving institutional integrity and equal treatment during the first semester on campus [27]. Ash and Schreiner claimed that students’ perceived institutional integrity significantly contributes to the success of colored students [22]. Based on the above literature review and taking into account the institutional integrity of private universities in China in educational practice, this study proposes the following hypothesis:

**H2:** Institutional integrity in China’s private universities has a positive impact on student thriving.

Prior research has validated that students’ perception of their institution is indicative of their feeling of belonging on campus [28]. For instance, students engage in campus integration when they perceive the institution’s honesty and dedication to their welfare, resulting in favorable psychological experiences [29]. Moreover, studies on student thriving have indicated that institutional integrity plays a vital role in fostering a pleasant psychological experience [22, 24]. The student’ sense of psychological community is the dependent variable in its relationship with institutional integrity and acts as an independent variable in the relationship with student thriving. We can assume that it indirectly mediates the association between institutional integrity and thriving. We formulated the following hypothesis:

**H3:** Institutional integrity in Chinese private universities positively affects students’ psychological sense of community.

The concept of growth mindset was first introduced by Dweck (2006) in the published book *Mindset*: *The New Psychology of Success* [30]. Specifically, a growth mindset is described as “a person’s belief that intelligence or abilities are easily influenced and can be developed through hard work, good strategies, and instruction from others” [31]. Research has confirmed that students possess a growth mindset as both a persuasive psychological trait and an intra-individual characteristic. Previous research has confirmed that growth mindset is both a persuasive personal psychological trait and an intrinsic character of students [32]. A growth mindset promotes positive educational outcomes because students with it tend to view challenges or difficulties as an integral part of their college experience and believe that any effort will pay off [31]. Students who possess a growth mindset are more inclined to persevere in their duties and actively participate in challenging learning activities [33], indicating more extraordinary achievements [34]. The moderating role of growth mindset is also supported by much of the research. For example, a study of 755 adolescents by Niu G. et al. has identified that having a growth mindset can moderate the relationship between adolescents’ exposure to cyberbullying and their likelihood of experiencing depression [35]. Lower-achieving students are likely to benefit significantly from growth mindset interventions as they become progressively more positively motivated and feel more resilient in the face of difficulties and failures [36]. Considering the challenging situations for students at private universities in China, it can be assumed that the increased level of students’ growth mindset likely reinforces the intensity of institutional integrity’s impact on student development. We then propose the following hypothesis:

**H4:** Student growth mindset in Chinese private universities positively moderates the impact of institutional integrity on student thriving.

Ryn and Patrick (2001) defined perceived teacher support as the extent to which students have confidence in the teacher’s worth and their bond with the teacher across four essential areas: emotional, instrumental, appraisal, and informational support [37]. Metheny et al. (2008) further argued that perceived teacher support pertains to the degree to which students employ teachers as readily available resources of assistance when they require help [38]. Perceived teacher support helps enhance academic motivation [39], has a significant impact on the learning process [40], creates a supportive learning environment [41], assists with academic adjustment, boosts higher degree aspirations [42], helps build confidence and make friends on campus [43], and increases the opportunities for success [44]. Vetter (2018) found that perceived teacher support accounted for 16% of the variability in the overall student thriving among 2,973 university students [45]. Students in Chinese private higher institutions tend to need to be stronger academically. However, they are very keen to complete their university studies successfully, and the support they receive from their teachers will be of enormous help to them [46, 47]. We propose the following hypothesis:

**H5:** In Chinese private universities, perceived teacher support positively affects student thriving.

According to McIntosh and Nelson (2012), teacher factors account for 36.1% of the variation in student’s psychological sense of community [13]. In a study conducted by Schreiner et al. (2013), a survey was administered to 2,889 students from various higher institutions [12]. The results revealed that teacher-related factors accounted for 22% of the variance in psychological sense of community and 32% of the variance in student thriving. Therefore, perceived support from teachers directly impacts their development and may also indirectly impact it through psychological sense of community. We propose the following hypothesis:

**H6:** Perceived support from teachers has a positive impact on the psychological sense of community among students in private universities in China.

Growth mindset is a persuasive psychological characteristic for individuals, and university students with growth mindset are more inclined to sense support from their teachers and maximize their education goals in university [48]. They hold the belief that their aptitudes are very susceptible to external influences and may be enhanced with assistance from others, notably their teachers [31]. Even though they meet some challenges, they may consider the efforts rewarding and see difficulties as part of university life [31]. Their anticipated outcomes are more likely to be achieved with a positive mindset [49]. The study conducted by Jiang et al. (2023) has confirmed that students’ growth mindset has a substantial impact on moderating the connection between perception of teacher support and their inclination to participate in self-regulated learning [50]. This study specifically focused on private universities in China. Thus, it can be assumed that the increased level of students’ growth mindset likely reinforces the intensity of perceived teacher support’s impact on student success. We formulate the following hypothesis:

**H7:** In Chinese private universities, students’ growth mindset positively moderates the impact of their perceived teacher support on student thriving.

This study aims to validate the mediating role of psychological sense of community and the moderating role of growth mindset in the effects of institutional integrity and perceived teacher support on student thriving in Chinese private universities. It combines the concept of holistic education in higher education with the Thriving Model, taking into account the current situation at private universities in China. We propose seven research hypotheses based on a review of relevant literature.

## 2. Method

### 2.1 Participants and procedure

In this study, data were collected by means of an online questionnaire because the advantages of the online questionnaire approach are that rich data can be collected in a shorter period of time, and data entry and processing are more convenient and accurate [51]. This study has received approval on human research protection from the Human Research Ethics Committee, Swan College, Central South University of Forestry and Technology, which fully complies with the principles expressed in the Declaration of Helsinki. The research targeted students pursuing bachelor’s degrees at four private universities in China. The study objectives of the online questionnaire and the link to the questionnaire were mostly distributed to the questioned students via their online social channels, such as WeChat, QQ, or personal email. Prior to the students completing the questionnaires, their consent as participants was informed and obtained in written form. The investigation was conducted from February 28, 2024, to March 15, 2024. After eliminating unqualified or invalid questionnaires using SPSS software, a total of 1792 valid questionnaires were obtained, meeting the acceptable sample size criteria [52].

**Fig 1:**
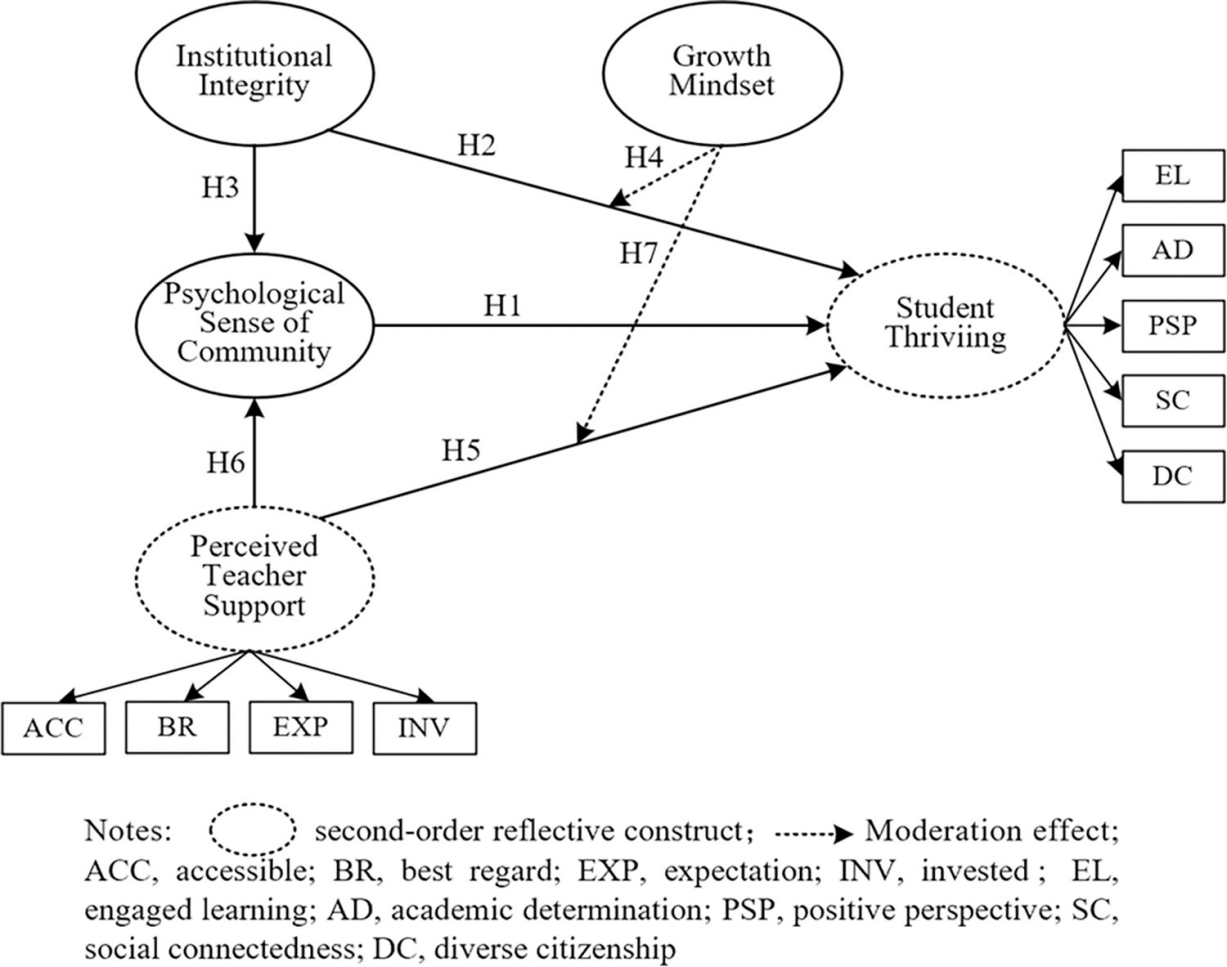
The hypothesized research model of the study. Notes: Dashed circle second-order reflective construct; Dashed line Moderation effect; ACC, acceiiible; BR, best regard; EXP, expectation; INV, invested; EL, engaged learning; AD, academic determination; PSP, positive perspective; SC, social connectedness; DC, diverse citizenship.

A partial least squares structural equation modeling (PLS-SEM) approach was utilized for this research. The PLS method, a causal-predictive approach to SEM, tests the predictive capabilities of a model whose structure stems from theory and logic. In this research, based on the reviews of some related theories and variables, the hypothesized model was proposed, which included hypotheses (Fig 1). This study utilized the PLS-SEM approach to gain an understanding of the strength of correlational connections in the hypothesized model. It also examined the direct, indirect, and total impacts of these variables on one another in the model. The study aimed to measure the mediating effect of psychological sense of community and the moderating role of growth mindset on the pathways from university support to thriving for undergraduates in private universities in China. The study also sought to define the strength of the relationships between these variables.

### 2.2 Measuring instruments and analysis method

The online survey questionnaire was split into two sections. The initial section of the questionnaire is to gather demographic data from the participating students, including gender, class, and college grades. The second section of the questionnaire consisted of detailed questions regarding five variables in the research model. The survey was carried out using a seven-point Likert scale, where each item was rated on a scale from “*1 = Strongly Disagree*” to “7 = Strongly Agree.” The questionnaire items utilized in this study were derived from the existing literature and research. The investigation questionnaire was distributed to five relevant experts to evaluate their appropriateness and provide advice prior to conducting the practical survey. We revised and modified these items based on the experts’ suggestions to align with the current research context.

#### 2.2.1 Student thriving

The Thriving Quotient developed by Schreiner (2010) was adopted in this study to measure the reflective construct of student thriving [11]. The three domains of student thriving were measured in five sub-constructs with 25 items. The first domain of academic thriving includes engaged learning and academic determination with 11 questions, such as “I feel as though I am learning things in my classes that are worthwhile to me as a person.” The second domain of interpersonal thriving involves social connectedness and diverse citizenship, and it involves 12 questions, such as “Other people seem to make friends more easily than I do.” The last psychological thriving is measured by one factor: positive perspective, with two questions like “My perspective on life is that I tend to see the glass as ‘half full’ rather than ‘half empty’”.

#### 2.2.2 Perceived teacher support

The reflective construct of perceived teacher support is measured by four composites originally developed by Metheny et al. (2008), which include invested, positive regard, expectation, and accessible [38]. In “invested,” there are eight items, such as “My teachers expect me to work hard at school.” There are five questions in “positive regard,” such as “My teachers push me to gain good academic achievement.” In “expectation,” five questions were asked, such as “My teachers enjoy having me as their student,” and in “accessible,” there are 3 questions, such as “My teachers will listen if I want to talk about a problem in my study”.

#### 2.2.3 Psychological sense of community

This study adopted the scale of psychological sense of community designed by Schreiner (2015), which consists of 4 questions, such as “I feel like I belong here” [53].

#### 2.2.4 Institutional integrity

Ash and Schreiner (2016) developed the scale of institutional integrity used in this study. It has three questions on the scale, such as “My experiences on this campus so far have met my expectations” [22].

#### 2.2.5 Growth mindset

The growth mindset scale adopted in this study is from the research instrument of Mesler et al. (2021). It includes four items, including “My intelligence is something that I can’t change very much” [54].

The proposed theoretical model underwent analysis using the Smart PLS 4.0 software. The assessment of the measurement model in this study primarily involved evaluating the internal consistency and reliability of the items, as well as the convergent validity and discriminant validity of the constructs. The structural model was evaluated using a bootstrap procedure with 5000 samples and a blindfolding procedure with ten omitted distances. When evaluating the inner model, some data were obtained through the three assessing stages: (1) collinearity issues, (2) significance and relevance of the structural model, and (3) explanatory and predictive power of the structural model. Estimates of structural model relationships (i.e., path coefficients) were obtained by running the PLS-SEM algorithm, and these values reflect the hypothesized relationships between the variables in the model. By interpreting these results, the critical relevant constructs were identified to explain the endogenous latent variables in the structural model of this research.

## 3. Results

### 3.1 Descriptive analysis

A total of 1792 valid responses were collected from students attending four private universities in China. These universities were ranked in the first, second, third, and fourth quartiles among Chinese private universities [5]. Table 1 displays the demographic information of the students who participated in the survey. They ranged from the first-year (freshman) to the fourth-year (junior). Of these students, 944 were female and 848 were male. 91.3% of the students surveyed were 18–22 years old, while only 8.7% were under 18 or over 22. Among these responses, their self-reported college grades mainly range from “mostly A’s and B’s” to “mostly C’s,” with only 5.8 and 13% presenting “below C average” and” primarily A’s,” respectively.

**Table 1:** Participants’ demographics.

Measure	Items	Number	Percentage (%)
Total Valid Respondents		1792	100
Gender	Male	944	57.3
	Female	848	52.7
Age	>22	65	3.6
	18–22	1636	91.3
	<18	91	5.1
Class	Freshman	656	36.6
	Sophomore	491	27.4
	Junior	425	23.7
	Senior	220	12.3
College Grade	mostly A’s	233	13
	mostly A’s and B’s	380	21.2
	mostly B’s	448	25
	mostly B’s and C’s	394	22
	mostly C’s	233	13
	below a C average	104	5.8

### 3.2 Measurement model assessment

The measurement model of this research was primarily assessed by examining the internal consistency and reliability of each item, as well as the values of convergent and discriminant validity. Additionally, we tested the degree of common method variance (CMV).

#### 3.2.1 Internal consistency, reliability, and convergent validity

This study utilized the PLS-SEM method to assess the internal consistency and reliability of the external model. This is accomplished by measuring the loading of the questions, the values of Cronbach’s alpha, and the composite reliability of the constructs. The recommended threshold for factor loadings is a minimum of 0.6 [55], while for Cronbach’s alpha, it is a minimum of 0.8 [56]. Additionally, the composite reliability (CR) values for each construct should exceed 0.7 [57]. Convergent validity, as defined by Mehmood and Najmi (2017), is the degree to which the measurements of the variables accurately assess the underlying theoretical construct because they share the exact range of variance [58]. Aside from assessing factor loading values and composite reliability, confirming convergent validity requires verifying that the average variance extracted (AVE) exceeds 0.5 [59].

Table 2 presents the assessed values of the measurement model of this study, including factor loadings, Cronbach’s alpha, composite reliability, and average AVE. The findings indicated that the factor loading values varied from 0.664 to 0.912, surpassing the essential threshold of 0.6. Additionally, Cronbach’s alpha values ranged from 0.813 to 0.942, beyond the recommended critical value of 0.8. Furthermore, the CR values varied from 0.863 to 0.958, beyond the threshold of 0.7, while the AVE values ranged from 0.554 to 0.885, surpassing the barrier of 0.5. The findings shown in Table 3 confirm the internal consistency, reliability, and excellent convergent validity of the measurement model.

**Table 2:** Reliability and validity results.

Constructs	Items	Factor Loading	Cronbach’s Alpha	rho_A	Composite Reliability	AVE
GM	GM1	0.836	0.925	0.926	0.944	0.770
	GM2	0.884				
	GM3	0.906				
	GM4	0.894				
	GM5	0.867				
PSC	PSC1	0.847	0.901	0.904	0.931	0.771
	PSC2	0.899				
	PSC3	0.908				
	PSC4	0.901				
ACC	ACC1	0.859	0.942	0.942	0.958	0.885
	ACC2	0.848				
	ACC3	0.846				
INV	INV1	0.801	0.935	0.937	0.947	0.69
	INV2	0.819				
	INV3	0.847				
	INV4	0.867				
	INV5	0.802				
	INV6	0.831				
	INV7	0.846				
	INV8	0.809				
BR	BR1	0.874	0.924	0.924	0.943	0.767
	BR2	0.869				
	BR3	0.844				
	BR4	0.850				
	BR5	0.884				
EXP	EXP1	0.852	0.938	0.938	0.953	0.801
	EXP2	0.873				
	EXP3	0.893				
	EXP4	0.871				
	EXP5	0.865				
INT	INT1	0.834	0.935	0.937	0.951	0.795
	INT2	0.912				
	INT3	0.880				
EL	EGL1	0.808	0.813	0.831	0.863	0.657
	EGL2	0.858				
	EGL3	0.802				
	EGL4	0.774				
AD	AD1	0.773	0.847	0.851	0.887	0.554
	AD2	0.664				
	AD3	0.800				
	AD4	0.826				
	AD5	0.772				
	AD6	0.745				
PSP	PSP1	0.869	0.899	0.9	0.926	0.713
	PSP2	0.851				
SC	SC1	0.792	0.912	0.913	0.932	0.696
	SC2	0.875				
	SC3	0.855				
	SC4	0.850				
	SC5	0.841				
	SC6	0.789				
DC	DC1	0.823	0.864	0.878	0.899	0.602
	DC2	0.861				
	DC3	0.772				
	DC4	0.813				
	DC5	0.770				
	DC6	0.784				

Notes: GM, growth mindset; PSC, psychological sense of community; ACC, accessible; BR, best regard; EXP, expectation; INV, invested; INT, institutional integrity; EL, engaged learning; AD, academic determination; PSP, positive perspective; SC, social connectedness; DC, diverse citizenship; AVE, average variance extracted. PTS and THR are second-order reflective constructs, so there is no estimation here.

**Table 3:** Fornell-Larcker criterion.

	GM	INT	ACC	EXP	INV	BR	PSC	AD	DC	EL	PSP	SC
GM	**0.878**											
INT	0.525	**0.893**										
ACC	0.770	0.536	**0.951**									
EXP	0.737	0.619	0.853	**0.909**								
INV	0.752	0.674	0.835	0.840	**0.851**							
BR	0.719	0.662	0.803	0.908	0.820	**0.897**						
PSC	0.627	0.801	0.653	0.689	0.753	0.722	**0.889**					
AD	0.544	0.550	0.570	0.607	0.661	0.634	0.553	**0.778**				
DC	0.604	0.673	0.639	0.664	0.702	0.686	0.663	0.643	**0.796**			
EL	0.471	0.464	0.476	0.527	0.562	0.540	0.472	0.682	0.538	**0.828**		
PSP	0.582	0.528	0.528	0.556	0.590	0.596	0.557	0.703	0.630	0.499	**0.858**	
SC	0.571	0.617	0.556	0.603	0.645	0.629	0.578	0.640	0.718	0.459	0.707	**0.847**

Notes: Diagonal elements in bold are the square root of the AVE. GM, growth mindset; psychological sense of community; ACC, accessible; BR, best regard; EXP, expectation; INV, invested; INT, institutional integrity; EL, engaged learning; AD, academic determination; PSP, positive perspective; SC, social connectedness; DC, diverse citizenship; AVE, average variance extracted. PTS and THR are second-order reflective constructs, so there is no estimation here.

#### 3.2.2 Discriminant validity

In order to evaluate the measurement model for this study, it was necessary to examine the discriminant validity of each construct. Discriminant validity refers to the degree to which two variables can be distinguished from each other through empirical testing. Two primary methods were utilized to assess it: the Fornell-Larcker criterion and the Heterotrait-Monotrait ratio (HTMT). The criterion for assessing the Fornell-Larcker criterion is that the square root of the average variance extracted (AVE) for each construct must be greater than its bi-variate association with other constructs [59]. Simultaneously, the HTMT threshold must be less than 0.90 [59]. The results showed that both the Fornell-Larcker criterion values (see Table 3) and the HTMT values between the constructs (see Table 4) satisfied the recommended criteria, hence confirming the discriminant validity of variables in this measurement model.

**Table 4:** Heterotrait-Monotrait ratio (HTMT).

	GM	INT	ACC	EXP	INV	BR	PSC	AD	DC	EL	PSP	SC
GM												
INT	0.562											
ACC	0.822	0.567										
EXP	0.788	0.656	0.901									
INV	0.804	0.715	0.881	0.840								
BR	0.770	0.705	0.851	0.863	0.877							
PSC	0.684	0.865	0.703	0.742	0.811	0.781						
AD	0.606	0.608	0.627	0.668	0.729	0.702	0.620					
DC	0.671	0.740	0.702	0.725	0.766	0.750	0.738	0.730				
EL	0.533	0.517	0.531	0.588	0.628	0.604	0.537	0.794	0.621			
PSP	0.633	0.570	0.568	0.598	0.636	0.645	0.610	0.789	0.698	0.566		
SC	0.618	0.663	0.595	0.646	0.693	0.677	0.630	0.713	0.794	0.517	0.771	

Notes: GM, growth mindset; PSC, psychological sense of community; ACC, accessible; BR, best regard; EXP, expectation; INV, invested; INT, institutional integrity; EL, engaged learning; AD, academic determination; PSP, positive perspective; SC, social connectedness; DC, diverse citizenship. PTS and THR are second-order reflective constructs, so there is no estimation here.

#### 3.2.3 Common method variance

Obtaining information about respondents’ perceptions by collecting self-rating scales in empirical studies may generate common method variance (CMV), thus affecting the results of the study. We took precautions in the survey to minimize the possible adverse effects of CMV. Furthermore, we employed an anonymity method in the survey, intentionally concealing the purpose behind each construct’s questions. Moreover, the variable results demonstrated adequate construct validity, suggesting that CMV did not have a substantial negative effect on the results of the study (see Table 5).

**Table 5:** Path coefficients.

Hypotheses	Relationship	Original Sample	Standard Deviation	T Statistics	Decision
H1	PSC →STR	0.153**	0.053	2.871	Supported
H2	INT→STR	0.441***	0.036	12.131	Supported
H3	INT→PSC	0.806***	0.017	46.523	Supported
H5	PTS→STR	0.450***	0.061	7.339	Supported
H6	PTS→PSC	0.677***	0.035	19.235	Supported

Notes: STR, Student Thriving; PSC, Psychological Sense of Community; GW, growth mindset; PTS, Perceived Teacher Support; INT, Institutional Integrity

***P < 0.001

**P< 0.01.

The degree of CMV was assessed using Harman’s one-factor test [60]. According to the exploratory factor analysis of the 54 questions in this survey, the explanatory variance for the first factor was 41.26%, less than the 50% criterion. This is a non-integrated factor, implying that CMV had little impact on the research.

### 3.3 Structural model assessment

**Fig 2:**
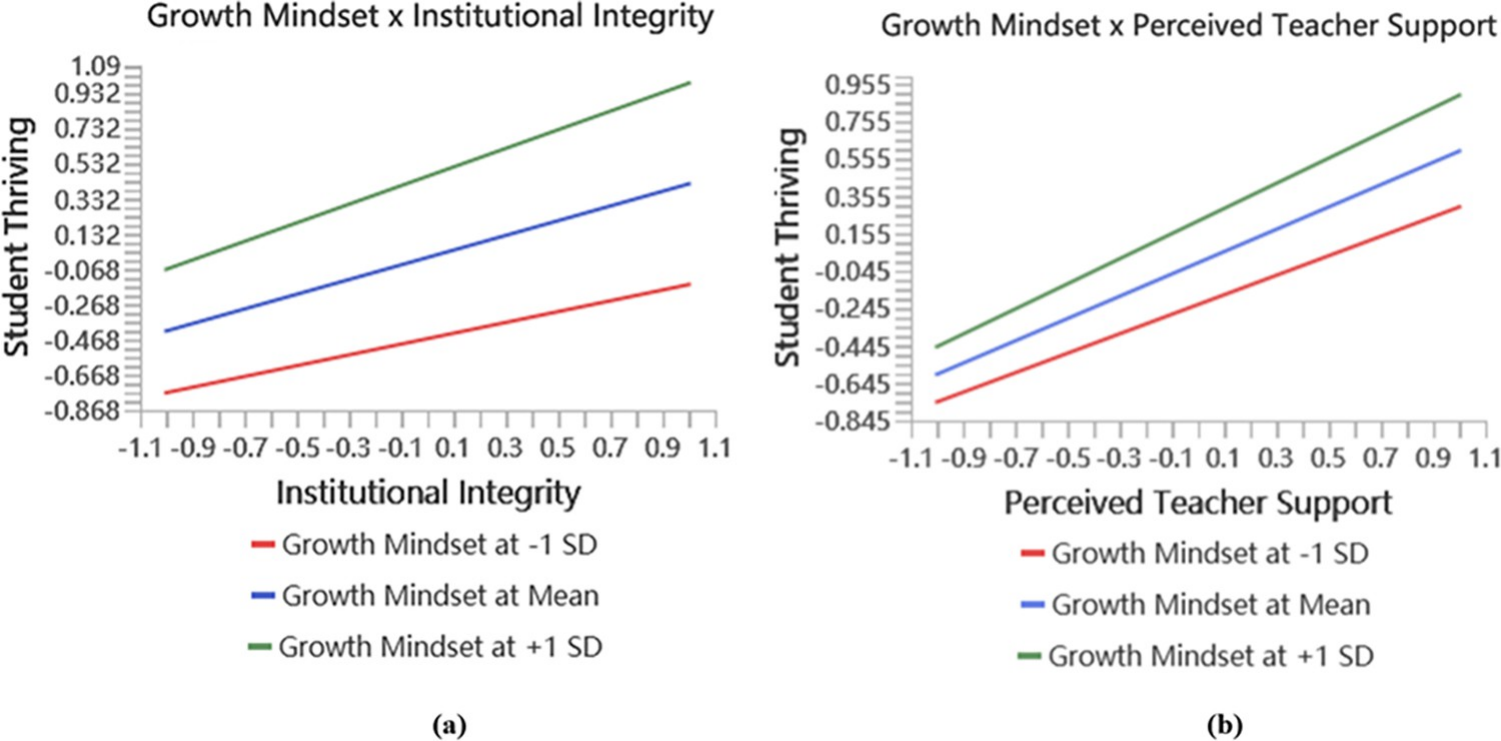
The moderating effect of growth mindset.

**Fig 3:**
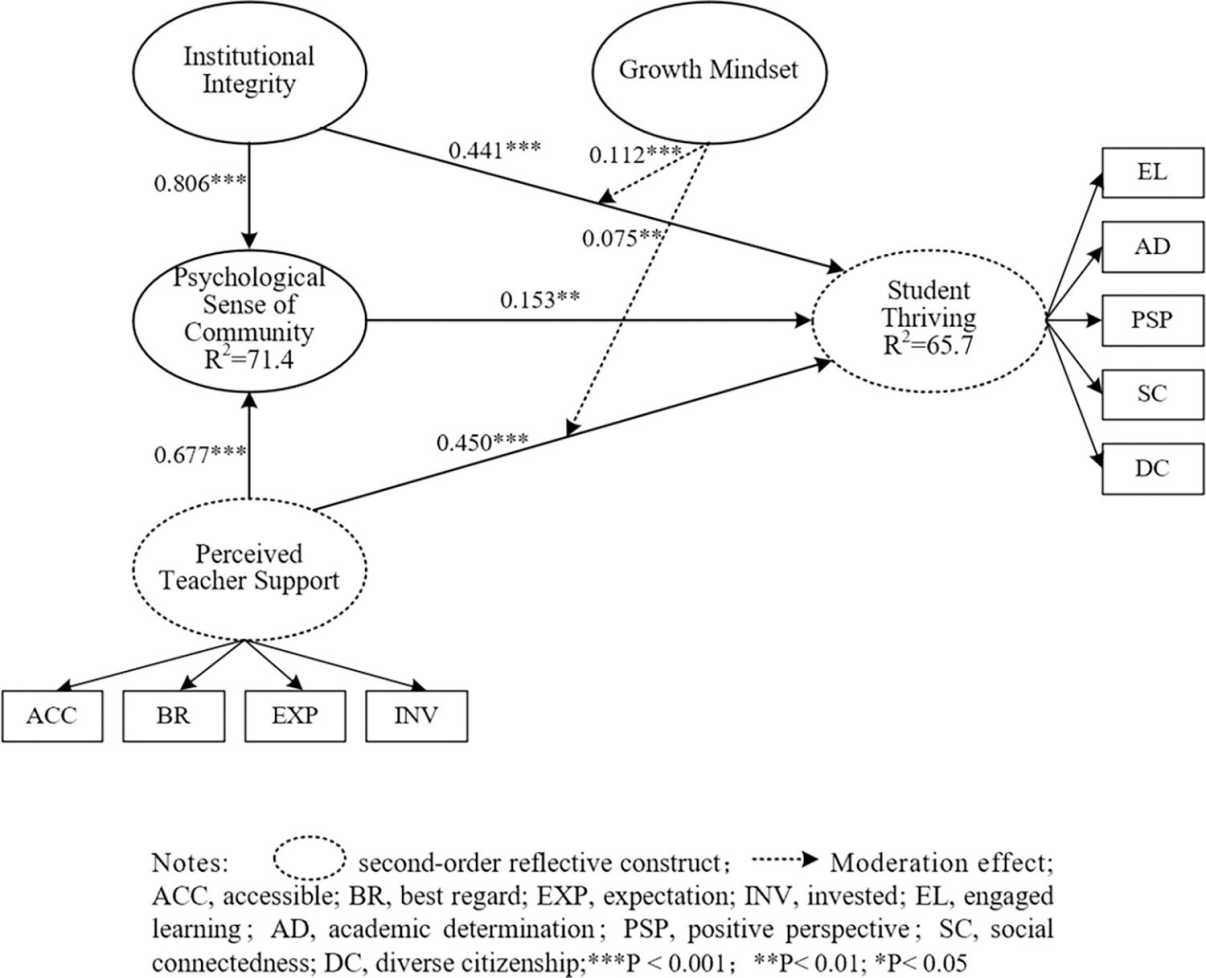
The significance of the inner model and the results of the path coefficient. Notes: Dashed circle second-order reflective construct; Dashed line Moderation effect; ACC, accessible; BR, best regard; EXP, expectation; INV, invested; EL, engaged learning; AD, academic determination; PSP, positive perspective; SC, social connectedness; DC, diverse citizenship;***P <0.001; **P< 0.01; *P< 0.05.

The structural model was evaluated using the standard model estimate, the bootstrapping procedure, and the PLS-predicting process. The findings are displayed in Tables 5, 6, and Figs 2 and 3.

**Table 6:** Mediation and moderation effects testing.

Relationship		Original Sample	Standard Deviation	T Statistics	Decision
INT→PSC→STR		0.210***	0.055	3.851	
PTS→PSC→STR		0.103*	0.038	2.739	
H4	GM × INT→STR	0.112***	0.031	3.628	Supported
H7	GM × PTS→STR	0.075**	0.025	3.043	Supported

Notes: STR, Student Thriving; PSC, Psychological Sense of Community; GW, growth mindset; PTS, Perceived Teacher Support; INT, Institutional Integrity

***P < 0.001

**P< 0.01

*P< 0.05.

#### 3.3.1 Collinearity issues

The structural model was assessed for collinearity issues by analyzing the VIF values of all predictor constructs in the model. The VIF values in the predictor constructs should be below five and preferably below a value of three to ensure that collinearity has no substantial effect on the structural model estimates [61]. The results indicated that all VIF values are within the range of 1.017 to 3.709 and are below the threshold of 5. Therefore, we can infer that the presence of collinearity among the predictor constructs is not a significant concern in this structural model, and the analysis of the left results report can proceed.

#### 3.3.2 Significance and relevance of the structural model

The significance and relevance of the structural model relationships were evaluated by the process of bootstrapping 10,000 samples. The beta coefficient (β), t-value, and p-value were derived to validate the significance of path coefficients. The findings shown in Table 5 demonstrate that all of the hypotheses have been confirmed. Additionally, the mediating and moderating effects have been examined and are displayed in Table 6. Consistent with hypothesis 1, the findings suggest that psychological sense of community (β = 0.153, t = 2.871) has a moderately positive effect on thriving. Consistent with H2, institutional integrity (β = 0.441, t = 12.131) positively impacts student thriving. Furthermore, the findings show that institutional integrity (β = 0.806, t = 46.532) positively influences psychological sense of community. Thus, H1, H2, and H3 are confirmed. Besides, the findings show that perceived teacher support positively influences thriving (β = 0.450, t = 7.339) and psychological sense of community (β = 0.0677, t = 19.235). Hence, H5 and H6 are supported (see Table 5). Path analysis was conducted to assess the significance of the mediating influence between the constructs in the research model. This analysis provided estimated p-values. The findings validated that psychological sense of community played a constructive role in mediating the impact of the independent variables, namely institutional integrity and perceived teacher support, on the dependent variable, student thriving. This is illustrated in Table 6.

The results of indirect effects show that the growth mindset moderates the impact of institutional integrity (β = 0.112, *t* = 3.628) and perceived teacher support (β = 0.075, *t* = 3.043) on student thriving. In addition, it is important to focus on the *f^2^* effect size of the interaction effect in order to verify the moderating influence of growth mindset on the impact of institutional integrity and perceived teacher support on student thriving. The *f^2^* effect size of the interaction effect is calculated, as the *f^2^* effect size indicates how much the moderation contributes to explaining the endogenous construct [61].This approach allows for the evaluation of the significance of the moderating effect. The values 0.005, 0.01, and 0.025 are considered to be appropriate benchmarks for small, medium, and large effect sizes, respectively [62]. Moreover, the calculated results of the *f^2^* moderating effect on the impact of institutional integrity and perceived teacher support on student thriving are 0.025 and 0.017, which mean large and medium effect sizes, respectively. In addition, this study created an interactive map (Fig 2) to better investigate the moderating effects. In order to conduct a more detailed analysis of the moderation effects, an additional simple slope was examined. This slope was used to visualize the strength of the linear association between institutional integrity and student thriving, as well as between perceived teacher support and student thriving, at both high and low levels of growth mindset (computed as +1 and −1 standard deviations from the mean). The results indicate that when the growth mindset is high, institutional integrity and perceived teacher support positively affect student thriving. Furthermore, institutional integrity and perceived teacher support have a lower positive impact on student thriving with a lower growth mindset. Thus, the results confirmed that the growth mindset increases the strength of the impact of institutional integrity and perceived teacher support on student thriving, further confirming H4 and H7 in the study.

#### 3.3.3 Explanatory and predictive power of the structural model

The PLS-SEM algorithm was run to assess the model’s explanatory power. The R^2^ values of the endogenous latent variables in the path model were analyzed to determine the extent to which they explain the variance of the endogenous constructs in the structural mode. The R^2^ results show that this model explains 71.4% of the variance in the psychological sense of community and 65.7% of thriving. The measured R^2^ values in this study are deemed acceptable based on the recommended values of R^2^ by Chin [57]. In addition, using the same method, we calculated the effect sizes *f*^*2*^ for all combinations of endogenous constructs and matching exogenous constructs in the structural model. Guidelines for analyzing *f*^*2*^ indicate that values of 0.02, 0.15, and 0.35 correspond to small, medium, and large effects, respectively, of the exogenous latent variable [63]. The effect size values in this model are all greater than 0.02, indicating a highly measurable effect. Using the PLS-predict procedure, we assessed the model’s predictive power. Q-square (Q^2^) was obtained through this computing process, indicating that the PLS path model performs better than the most naïve benchmark. The Q-square values for thriving (Q^2^ = 0.286) and psychological sense of community (Q^2^ = 0.526) are all greater than zero, indicating that the model has a satisfactory level of predictive power.

The assessment of the structural model confirmed the validity of all hypotheses in the study (see Fig 3). The results of the study suggested that psychological sense of community plays a crucial role in mediating the impacts of institutional integrity and perceived teacher support on student thriving. Growth mindset positively moderates the impacts of institutional integrity and perceived teacher support on student thriving.

## 4. Discussions

### 4.1 Psychological sense of community among students in Chinese private universities positively affects student thriving

The study’s findings support the initial hypothesis that psychological sense of community (β = 0.153, *t* = 2.871) positively influences student thriving (H1). The findings corresponded with prior research concerning the role of this psychological characteristic as a contributor to student thriving [12, 15]. The psychological experience of college students on campus can contribute significantly to their academic success, personal development, or future civic engagement [11], and students from private universities in China tend to be more psychologically dependent [23]. When these students living and studying on the campus of private universities feel a sense of belonging and think they matter to their university, they will be motivated to participate in various activities and interact regularly with their peers, teachers, and tutors. Through this, these students improve their social connectedness with the people around them, greatly enhancing their interpersonal abilities. Similarly, their sense of ownership, emotional connections with their peers, and interdependent partnerships will inspire them to engage more in their learning and take advantage of their strengths to achieve their educational goals. Thus, the psychological sense of community for students from private universities can have a direct relationship with their social connectedness, academic achievements, and psychological health.

### 4.2 Institutional integrity in China’s private universities has a positive impact on student thriving and psychological sense of community

According to the research results, institutional integrity also influences student thriving positively (β = 0.441, *t* = 12.131), which is in line with H2. The extent to which institutional practices reflect its mission is an indicator of students’ satisfaction [26], students’ personal development and academic achievements on campus [4], and their adjustment and engagement in the university community [24]. Consequently, when students from private universities find that their expectations for their university life have been met through campus experiences and they have received equal treatment regardless of their background, they may feel that they belong on campus and build a positive attitude toward college life. Once they are satisfied with their life in these private universities, they not only put much effort into their learning, but they are also willing to make friends and actively contact their teachers, resulting in positive psychological experiences at the same time [29].

The results indicated that institutional integrity has a positive effect on students’ psychological sense of community (β = 0.806, *t* = 46.523), per H3 of the study. According to previous research, institutional integrity reflects students’ perceptions of “the degree of congruence between the espoused mission and goals of a college or university and the actions of administrators, faculty, and staff” [25]. Students at private universities in China do not have the opportunity to attend public ones. Thus, they have higher expectations of the universities they attend. Suppose the private university can keep promises to the missions and goals reflected through their policy and daily educational practice. In that case, students will better integrate into the campus environment and have positive psychological experiences. It indicates that students’ psychological experiences mediate the relationship between institutional integrity and student thriving. In China’s private universities, a high level of institutional integrity bodes well for students’ solid sense of community, which in turn promotes student thriving [64].

### 4.3 Perceived teacher support positively affects student thriving and their psychological sense of community in Chinese private universities

The research findings revealed that perceived teacher support in private universities in China has a positive impact on students’ psychological experiences (β = 0.0677, *t* = 19.235) and their thriving (β = 0.450, *t* = 7.339), which supports the previous hypotheses in the study (H5 and H6).

Perceived teacher support encompasses the level of trust students have in teachers’ values, their rapport with teachers [51], and the amount to which students perceive their teachers as available resources when they require help [53]. In contrast, students from private universities in China tend to feel unsatisfied with or not confident about their academic achievement or personal development in college [65]. Support from teachers can function as an educational context for these students, and it strongly connects with students’ education on campus. During their time in private universities, if students can sense the caring, trust, and empathy from their teachers, tutors, or advisors, or if their teachers can provide timely assistance and help solve problems, they may feel valued in university. Thus, in turn, they can improve their emotional connections with others on campus, thereby creating a supportive environment for them. Consequently, it affects not only students’ inner lives but also their beliefs connected with them, improving a much higher sense of belonging and, in turn, helping them better explore their university education. Once a safe learning environment with teacher support is created, not only their academic motivation, learning attention, and college outcomes can be enhanced, but they are also ready to make friends and get involved in various activities, obtaining abundant positive psychological experiences through college life [39, 43]. Thus, student thriving, including academic, interpersonal, and psychological aspects, can be improved through their perceived teacher support.

The findings align with earlier research, suggesting that a significant amount of perceived teacher support is a reliable indicator of a robust sense of community on campus, which in turn promotes student thriving. Teachers’ factors have a direct and indirect impact on the thriving of students in private universities, specifically through the psychological sense of community.

### 4.4 Growth mindset positively moderates the impacts of institutional integrity and perceived teacher support on student thriving in Chinese private universities

The research results corresponded with the hypotheses in this study (H4 and H7). Growth mindset moderates the impact of institutional integrity (β = 0.112, *t* = 3.628) and perceived teacher support (β = 0.075, *t* = 3.043) on student thriving in Chinese private higher institutions.

A student’s growth mindset is confirmed as an intra-individual characteristic [32], which means “a person’s belief that intelligence or abilities are easily influenced and can be developed through hard work, good strategies, and instruction from others” [31]. Students at Chinese private universities often need more confidence or are dissatisfied with their achievements because they failed to attend a public university [46]. A growth mindset, as an intrinsic personality trait, is not something everyone has. Suppose students attending private institutions hold the belief that their intelligence or abilities can be easily altered and enhanced by diligent effort, effective strategies, or guidance from others. Under such circumstances, there is a high probability of achieving numerous positive educational outcomes. For example, they can optimize their educational goals even if they encounter challenges or difficulties when attending private universities. The research has confirmed the positive effects of institutional integrity and perceived teacher support on student comprehensive development academically, socially, and psychologically. High levels of growth mindset in students from private universities can increase the strength of these positive effects. These students with high levels of growth mindset exhibit increased persistence. They are involved in challenging tasks, even when they cannot perceive very high levels of institutional integrity or support from teachers at these private universities for various reasons. Growth mindset acts as a moderator for these students to obtain more extraordinary educational achievement.

## 5. Limitations of the study and further development

Several limitations should be stated when discussing the results of this research, aiming to explore potential future research directions building upon this current work. Firstly, self-reported questionnaires were adopted in the study, and reasons like social desirability in this kind of survey may influence the collected data. Furthermore, this study was carried out using a quantitative approach, specifically through the administration of a questionnaire survey. Hence, in the future, qualitative research methods such as interviews and observations can be employed to delve deeper into this research issue. Thirdly, as this study only focuses on students pursuing bachelor’s degrees at four private universities in China, the generalizability of the findings reflects the limitations of this study. Therefore, future research endeavors might prioritize examining student samples from diverse educational levels or systems, as well as those from various cultural backgrounds, in order to enhance the generalizability of the research findings on this topic.

Besides, given the research results about the psychological characteristics in this study, it is advisable to validate their indirect effect between other contributing variables, i.e., campus culture, major certainly, etc., and student thriving in future research. We also recommend a deeper exploration of the influence pathways of these psychological traits, whether as dependent or independent variables, on student thriving. We can explore the role of psychological factors in students’ development on campus in a more profound and broader manner, which will further contribute to our understanding of student thriving.

## 6. Implications for future educational implementation

Since positive external interventions or environmental improvements can make a difference in students’ campus experience, thereby enriching their college experience and contributing to their achievement [66], interventions on students’ psychological experiences and university support improvements are advisable to achieve anticipated outcomes on campus for students at private universities. Administrators can investigate students’ campus experiences and solicit their feedback, including how they perceive institutional integrity and teacher support, as well as the degree of their psychological sense of community and level of growth mindset on campus. Following a comprehensive assessment, administrators should implement appropriate interventions and actions.

Some lectures, on-campus activities, and psychological training programs can be established based on students’ psychological interventions, helping them increase their feelings of membership, relationship, ownership, and partnership and embrace a growth mindset. Previous research has shown that students who initially perform poorly are likely to benefit significantly from psychological interventions by gradually becoming positively motivated and showing greater resilience in the face of academic failure [36]. Suppose the environmental support at some private universities is difficult to improve in a short period of time for some reason. In that case, administrators can attempt to enhance students’ sense of psychological community and growth mindset through various interventions, thereby enhancing the effects of students’ psychological factors on their thriving. Furthermore, the administrators of these private universities should shift their focus from the enrollment and employment rates of graduates to the student’s campus experience or comprehensive development. Although these institutions mainly operate on a self-financing basis, they should keep their admissions process manageable, keeping promises to the missions and goals reflected through their administration policy and educational practice. These institutions should also place a greater value on their teachers. As a result, students can have greater confidence in the connection between them and their teachers, as well as a strong conviction that their teachers are readily available support resources when they need help.

## 7. Conclusions

This study was carried out in the setting of Chinese private universities, using a PLS-SEM approach. The key finding is that psychological sense of community plays a crucial role in mediating the positive effects of institutional integrity and perceived teacher support on student thriving. The moderation roles of growth mindset in the effects of institutional integrity and perceived teacher support on student thriving were validated. This study contributes to the advancement of research on students’ psychological experiences on campus and their thriving. The urge is for educators, policymakers, and academics at private universities in China to enhance their understanding of the significance of students’ psychological experiences on campus. Some recommendations were also provided for the administrators, faculty, and staff members, as well as the related researchers, pushing them to create relevant interventions and support or do further research related to enhancing student thriving, improving the quality of their graduates, and facilitating the sustainable growth of private universities in China.

## Supporting information

S1 Data (SAV)
